# Metaformer: A Transformer That Tends to Mine Metaphorical-Level Information

**DOI:** 10.3390/s23115093

**Published:** 2023-05-26

**Authors:** Bo Peng, Yuanming Ding, Wei Kang

**Affiliations:** 1 Communication and Network Laboratory, Dalian University, Dalian 116622, China; 2 School of Information Engineering, Dalian University, Dalian 116622, China

**Keywords:** hierarchical attention, multi-head attention, graph neural networks, feature diversity, information interaction

## Abstract

Since introducing the Transformer model, it has dramatically influenced various fields of machine learning. The field of time series prediction has also been significantly impacted, where Transformer family models have flourished, and many variants have been differentiated. These Transformer models mainly use attention mechanisms to implement feature extraction and multi-head attention mechanisms to enhance the strength of feature extraction. However, multi-head attention is essentially a simple superposition of the same attention, so they do not guarantee that the model can capture different features. Conversely, multi-head attention mechanisms may lead to much information redundancy and computational resource waste. In order to ensure that the Transformer can capture information from multiple perspectives and increase the diversity of its captured features, this paper proposes a hierarchical attention mechanism, for the first time, to improve the shortcomings of insufficient information diversity captured by the traditional multi-head attention mechanisms and the lack of information interaction among the heads. Additionally, global feature aggregation using graph networks is used to mitigate inductive bias. Finally, we conducted experiments on four benchmark datasets, and the experimental results show that the proposed model can outperform the baseline model in several metrics.

## 1. Introduction

Originally designed to solve machine translation problems [1,2], the Transformer [3,4] model has been widely introduced into computer vision (CV) [5,6,7], natural language processing (NLP) [8], speech processing [9,10,11], audio processing [12,13], chemistry [14], and life sciences [15] due to its powerful modelling capabilities and applicability. It has contributed significantly to the development of these fields.

In computer vision, Convolutional Neural Networks (CNNs) [16,17,18] are traditionally used as the primary means of processing. Convolution is well suited for processing regular, high-dimensional data and allows for automatic feature extraction. However, convolution suffers from obvious localisation constraints. The conditional assumption is that points in the space are only associated with their neighbouring grids, whereas distant grids are not associated with each other. Although this limitation can be alleviated to some extent by expanding the convolution kernel, it still cannot solve the problem fundamentally. After introducing the Transformer, some researchers have tried to introduce the Transformer model architecture into the field of computer vision. Transformer has a larger field of perception than CNN, so it captures rich global information and can better understand the whole image. Ramachandran et al. [19] constructed a vision model without using convolution, which uses a full-attention mechanism instead of convolution to improve the localisation constraint in convolution. In addition, Transformer has shown excellent performance in other CV areas such as image classification [6,20], object detection [5,21], semantic segmentation [22], image processing [22], and video understanding [5].

Sequential data are more suitable for processing using Transformer than computer vision. In the traditional field of time series prediction, most of them rely on Recurrent Neural Network (RNN) [23,24] models, among which the more influential ones include Gated Recurrent Unit (GRU) [25] and Long Short-term Memory (LSTM) [26,27] networks. For example, Mou et al. [28] proposed a Time-Aware LSTM (T-LSTM) with temporal information enhancement, whose main idea is to divide memory states into short-term memory and long-term memory, adjust the influence of short-term memory according to the time interval between inputs (the longer the time interval, the smaller the influence of short-term memory), and then reorganise the adjusted short-term memory and long-term memory into a new memory state. However, the emergence of Transformer soon shook the dominance of RNN family models in the field of time series prediction because of the following bottlenecks of RNNs in dealing with long-time prediction problems.

(1) Parallelism bottleneck: The RNN family of models requires the input data to be arranged in temporal order and computed sequentially according to the order of arrangement. This serial structure has the advantage that it inherently contains the portrayal of positional relationships, but it also constrains the model from being computed in parallel. Especially when facing long sequences, the inability to parallelise means more time and cost.

(2) Gradient bottleneck [29]: One performance bottleneck of RNN networks is the frequent problem of gradient disappearance or gradient explosion during training. Most neural network models optimise model parameters by computing gradients. Gradient disappearance or gradient explosion can cause the model to fail to converge or converge too slowly, which means that for the RNN family of networks, it is difficult to make the model better by increasing the number of iterations or increasing the size of the network.

(3) Memory bottleneck: For each moment, the RNN network requires a positional input $x_{t}$ and a hidden input $h_{t-1}$, which will be fused within the model according to the inherent rules to produce a hidden state $h_{t}$. Therefore, when the sequence length is too long, the $h_{t}$ almost no longer contains the earlier positional input; that is, the “forgetting” phenomenon occurs.

Compared with the RNN family of models, Transformer portrays the positional relationships between sequences by positional encoding without recursively feeding sequential data. This processing makes the model more flexible and provides the maximum possible parallelisation for time series data. The positional encoding also ensures that no forgetting occurs. The information at each location has an equal status for the Transformer. Additionally, using an attention mechanism to extract internal features allows the model to choose to focus on important information. The problem of gradient disappearance or gradient explosion can be avoided by ignoring irrelevant and redundant information. Therefore, based on the above advantages of Transformer models, many scholars are now trying to use Transformer models for time series tasks.

## 2. Research Background

Transformer is a typical encoder-decoder-based sequence-to-sequence [30] model, and this structure is well suited for processing sequence data. Several researchers have tried to improve the Transformer model to meet the needs of more complex applications. For example, Kitaev et al. [31] proposed a Reformer model that uses Locality Sensitive Hashing Attention (LSH) to reduce the complexity of the original model from $O(L^{2})$ to O(Llog(L)). Zhou et al. [32] proposed an Informer model for Long Sequence Time Series Forecasting (LSTF), which accurately captures the long-term dependence between output and input and exhibits high predictive power. Wu et al. [33] proposed the Autoformer model, which uses a deep decomposition architecture and an autocorrelation mechanism to improve LSTF accuracy. The Autoformer model achieves desirable results even when the series is predicted much longer than the length of the input series, i.e., it can predict the longer-term future based on limited information. Zhou et al. [34] proposed the FEDformer model, which provides a way to apply the attention mechanism in the frequency domain and can be used as an essential complement to the time domain analysis.

The Transformer model described above focuses on reducing its temporal and spatial complexity, but needs to enhance the diversity of the information it captures. The attention mechanism is the core part of the Transformer used for feature extraction. It is designed to allow the model to focus on more important information, which means there is a certain amount of information loss. The multi-head attention mechanism can compensate for this. However, since each attention head captures similarly, there is no way to ensure that each attention head is capturing different vital features. Since the multi-head attention mechanism essentially divides multiple mutually independent subspaces, this approach completely cuts off the connection between each subspace, which leads to a lack of interaction between the information captured by multiple heads. Based on these problems, this paper proposes a hierarchical attention mechanism that features each layer using a different attention mechanism to capture features. The higher layers will use the information captured by the lower layers, thus enhancing the Transformer’s ability to perceive deeper information.

## 3. Research Methodology

### 3.1. Problem Description

Initially, the Transformer model was proposed by Waswani et al. to solve the machine translation problem, so Vanilla Transformer is more suitable for processing textual data. For example, the primary processing unit of the Vanilla Transformer model is a word vector, and each word vector is called a token. In contrast, in the time series prediction problem, our basic processing unit becomes a timestamp. If we want to apply Transformer to a time series problem, the reasonable idea is to encode the multivariate sequence information of each timestamp into a token vector. This modelling approach is also the treatment of many mainstream Transformer-like models.

Here, for the convenience of the subsequent description, we define the dimension of the token as *d*, the input length of the model as *I*, and the output length as *O*. Further, the model’s input can be defined as $X=\left\{x_{1},\cdots ,x_{I}\right\}\in R^{I\times d}$, and the model’s output as $\hat{X}=\left\{{\hat{x}}_{1},\cdots ,{\hat{x}}_{O}\right\}\in R^{O\times d}$. Therefore, this paper aims to learn a mapping T(·) from the input space to the output space.

(1) $$\hat{X}=T\left(X\right)$$

### 3.2. Model Architecture

**Figure 1:**
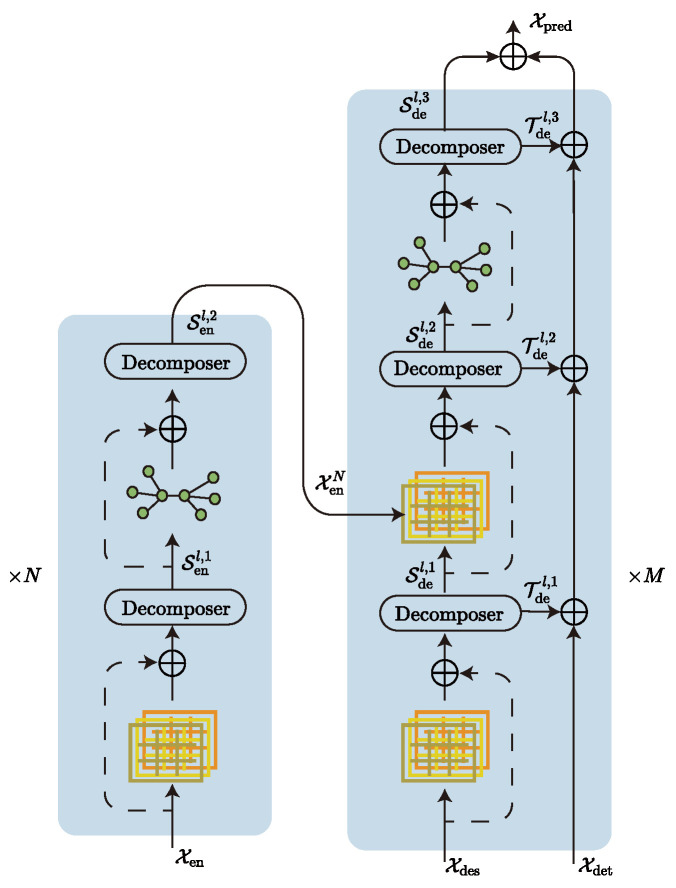
Model Body Structure.

Our model (Figure 1) continues the Transformer architecture in the main body, and we also added a decomposer to the model by referring to Autoformer’s sequence decomposition model. The function of the decomposer is to filter trend-cyclical and seasonal parts. The advantage is that removing trend parts from the series allows the model to focus better on the hidden periodic information of the series, and Wu et al. [33] have shown that this decomposition is effective. In addition, the model uses a coder–decoder structure, where the encoder is responsible for mapping the information from the input space to the feature space, and the decoder is responsible for mapping the information from the feature space to the target space. The model is a typical sequence-to-sequence model, since both the input and output of the model are sequence-type data. In addition, we try to use a hierarchical attention mechanism instead of the original multi-head attention mechanism and a graph network instead of the original feedforward neural network inside the codec, which can improve the diversity of captured information and the mitigate token-uniformity inductive bias [35,36] of the model, respectively.

#### 3.2.1. Decomposer

The main difficulty of time series forecasting lies in discovering the hidden trend-cyclical and seasonal parts information from the historical series. The trend-cyclical records the overall trend of the series, which has an essential influence on the long-term trend-cyclical of the series. The seasonal parts record the hidden cyclical pattern of the series, which mainly shows the regular fluctuation of the series in the short term. It is generally difficult to predict these two pieces of information simultaneously. The basic idea is to decompose the two, extracting the trend-cyclical from the sequence using average pooling and filtering the seasonal period using the trend-cyclical, which is how Decomposer implements the decomposed information, as shown in Algorithm 1.
**Algorithm 1** Decomposer**Require:**  X**Ensure:**  S,T  1:T←avgpool(padding(X))  2:S←X−T

Here, $X\in R^{L\times d}$ is the input sequence of length *L*. $T,S\in R^{L\times d}$ is the decomposed trend-cyclical and seasonal parts where the role of padding is to ensure that the decomposed series remains equal in dimension to the input sequence.

The decomposer module has a relatively simple structure. However, it can decompose the forecasting task into two subtasks, i.e., mining hidden periodic patterns and forecasting overall trends. This decomposition can reduce the difficulty of prediction to a certain extent and, thus, improve the final prediction results.

#### 3.2.2. Encoder

The encoder is mainly responsible for encoding the input data and realizing the transformation from the input space to the feature space. The decomposer in the encoder is more like a filter because, in the encoder, we focus more on the seasonal parts of the sequence and ignore the trend-cyclical. The input data are passed through a hierarchical attention layer for initial key feature extraction. After which, the decomposer extracts the seasonal part’s features in the sequence and they are further fed into the graph network to mitigate inductive bias. After stacking *N* layers, The seasonal parts features thus obtained will be auxiliary inputs to the decoder. Algorithm 2 describes the computation procedure.
**Algorithm 2** Encoder**Require:**  $X_{\mathrm{en}}$**Ensure:** $X_{\mathrm{en}}^{N}$  1:**for** l=1,⋯,N **do**  2:   **if** l=0 **then**  3:     $X_{\mathrm{en}}^{l-1}\leftarrow X_{\mathrm{en}}$  4:   **end if**  5:   $S_{\mathrm{en}}^{l,1},\_\leftarrow D\left(H\left(X_{\mathrm{en}}^{l-1}\right)+X_{\mathrm{en}}^{l-1}\right)$  6:   $S_{\mathrm{en}}^{l,2},\_\leftarrow D\left(G\left(S_{\mathrm{en}}^{l,1}\right)+S_{\mathrm{en}}^{l,1}\right)$  7:   $X_{\mathrm{en}}^{l}\leftarrow S_{\mathrm{en}}^{l,2}$  8:**end for**

Here, $X_{\mathrm{en}}\in R^{I\times d}$ denotes the historical observation sequence. *N* denotes the number of stacked layers of the encoder. $X_{\mathrm{en}}^{N}$ denotes the output of the *N*-th layer encoder. D denotes the decomposer operator. G denotes the graph network operator and H denotes the hierarchical attention mechanism, the concrete implementation of which will be described later.

#### 3.2.3. Decoder

The structure of the decoder is more complex than that of the encoder. However, its internal modules are identical to the encoder’s, but use a multi-input structure. It goes through two hierarchical attention calculations and three sequence decompositions in turn. Assuming that the model’s encoder is a feature catcher, the decoder is a feature fuser that fuses and corrects the inputs from different sources to obtain the correct prediction sequence. The decoder has three primary input sources: the seasonal parts $X_{\mathrm{des}}$ and the trend-cyclical $X_{\det}$ extracted from the original series, and the seasonal parts $X_{\mathrm{en}}^{N}$ captured by the decoder. The computation of the trend-cyclical and seasonal parts is kept relatively independent throughout the computation process. Only at the final output is a linear layer used to fuse the two to obtain the final prediction $X_{\mathrm{pred}}$. The computation process is described in Algorithm 3.
**Algorithm 3** Decoder**Require:**  $X_{\mathrm{en}},X_{\mathrm{en}}^{N}$**Ensure:**  $X_{\mathrm{pred}}$    1:$X_{\mathrm{ens}},X_{\mathrm{ent}}\leftarrow D\left(X_{\mathrm{en}\frac{I}{2}:I}\right)$    2:$X_{\mathrm{des}}\leftarrow X_{\mathrm{ens}}\|0_{0:\frac{I}{2}}$    3:$X_{\det}\leftarrow X_{\mathrm{ent}}\|{\bar{X}}_{0:\frac{I}{2}}$    4:**for** l=1,⋯,M **do**    5:    **if** l=1 **then**    6:         $X_{\mathrm{de}}^{l-1}\leftarrow X_{\mathrm{des}}$    7:       $T_{\mathrm{de}}^{l-1}\leftarrow X_{\det}$    8:    **end if**    9:    $S_{\mathrm{en}}^{l,1},T_{\mathrm{de}}^{l,1}\leftarrow D\left(H\left(X_{\mathrm{de}}^{l-1}\right)+X_{\mathrm{de}}^{l-1}\right)$  10:   $S_{\mathrm{de}}^{l,2},T_{\mathrm{de}}^{l,2}\leftarrow D\left(H\left(S_{\mathrm{de}}^{l,1},X_{\mathrm{en}}^{N}\right)+S_{\mathrm{de}}^{l,1}\right)$  11:   $S_{\mathrm{de}}^{l,3},T_{\mathrm{de}}^{l,3}\leftarrow D\left(G\left(S_{\mathrm{de}}^{l,2}\right)+S_{\mathrm{de}}^{l,2}\right)$  12:   $X_{\mathrm{de}}^{l}\leftarrow S_{\mathrm{de}}^{l,3}$  13:   $T_{\mathrm{de}}^{l}\leftarrow T_{\mathrm{de}}^{l-1}+W_{l,1}*T_{\mathrm{de}}^{l,1}+W_{l,2}*T_{\mathrm{de}}^{l,2}+W_{l,3}*T_{\mathrm{de}}^{l,3}$  14:**end for**  15:$X_{\mathrm{pred}}\leftarrow W_{S}*X_{\mathrm{de}}^{M}+T_{\mathrm{de}}^{M}$

Here, $X_{\mathrm{en}}$ denotes the original sequence, which is also the input to the encoder. It is decomposed into trend-cyclical and season parts $X_{\mathrm{ens}},X_{\mathrm{ent}}$ before feeding into the decoder as the initial input.

### 3.3. Hierarchical Attention Mechanism

The hierarchical attention mechanism, as the first feature capture unit of Metaformer, is at the model’s core and, therefore, has a significant impact on the subsequent work. Most Transformer-like models use the multi-head attention mechanism to complete the first step of feature extraction. However, the multi-head attention mechanism itself has significant drawbacks: (1) each head uses the exact attention mechanism, which cannot guarantee the diversity of captured information and may even miss some critical information. (2) Each head belongs to a separate subspace, and the lack of information interaction between heads is not conducive to the deep understanding of information by the model. Therefore, we propose a hierarchical attention mechanism for the first time. First, a hierarchical structure is used, where each layer uses a different attention mechanism to capture features separately, which ensures the diversity of information circulating in the network; second, a cascading interaction is used, where the information captured by the lower layer will be reused by the upper layer, which will deepen the depth of information understanding by the model. We know that when we humans understand language, we not only focus on the surface meaning of words, but can also understand the metaphors behind the words. Inspired by this, we use a hierarchical structure to model this phenomenon and, thus, improve the network’s ability to perceive information in three dimensions.

#### 3.3.1. Traditional Multi-Head Attention Mechanism

In the multi-head attention mechanism, only one type of attention computation scaled dot-product attention is used. The multi-head attention mechanism first takes as input three vectors of queries, keys, and values with $d_{m}$ dimension, and each head is projected to $d_{k},d_{k}$ and $d_{v}$ dimensions using a linear layer. The attention function is then computed to produce a $d_{v}$ dimensional output value. Finally, the output of each attention head is stitched together and passed through a linear layer to obtain the final output.

(2) $$H\left(Q,K,V\right)=L_{\theta_{o}}\left(\overset{h}{\underset{i=1}{∐}}A\left(L_{\theta_{q}}^{i}\left(Q\right),L_{\theta_{k}}^{i}\left(K\right),L_{\theta_{v}}^{i}\left(V\right)\right)\right)$$

Equation (2) calculates the multi-headed attention mechanism, where $L_{\theta_{q}},L_{\theta_{k}},L_{\theta_{v}},L_{\theta_{o}}$ denotes the linear layer with projection parameter matrix $W^{Q}\in R^{d_{m}\times d_{k}},W^{K}\in R^{d_{m}\times d_{k}},W^{V}\in R^{d_{m}\times d_{v}},W^{O}\in R^{hd_{v}\times d_{m}}$, respectively. *h* denotes the number of heads of attention. A denotes scaled dot-product attention. ∐ denotes sequential cascade.

#### 3.3.2. Hierarchical Attention Mechanism

**Figure 2:**
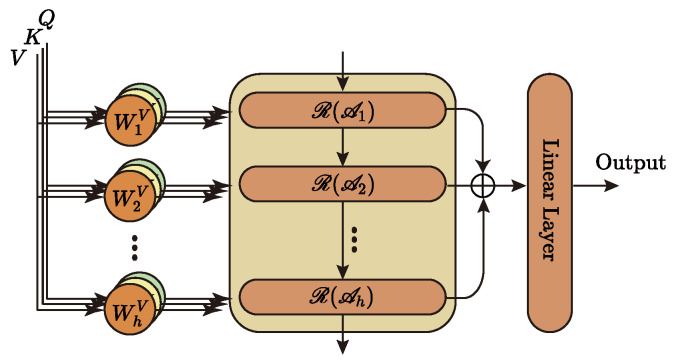
Hierarchical attention mechanism.

We propose a hierarchical attention mechanism to address the shortcomings in the multi-head attention mechanism, aiming to enhance the model’s deep understanding of the information. Figure 2 depicts the central architecture of the hierarchical attention mechanism, and Algorithm 4 describes its implementation.
**Algorithm 4** Hierachical Attention**Require:**  Q,K,V**Ensure:**  Y  1:**for** i=1,⋯,N **do**  2:   **if** i=1 **then**  3:     H←RandomInitialisation  4:   **end if**  5:   $H\leftarrow R\left(A_{i}\left(L_{\theta_{q}}^{i}\left(Q\right),L_{\theta_{k}}^{i}\left(K\right),L_{\theta_{v}}^{i}\left(V\right)\right),H\right)$  6:   Y←Y‖H  7:**end for**  8:$Y\leftarrow L_{\theta_{o}}\left(Y\right)$

Here, $L_{\theta_{q}},L_{\theta_{k}},L_{\theta_{v}},L_{\theta_{o}}$ has the same meaning as in Equation (2). R denotes the GRU unit. Y records the information of each layer and finally maps it to the specified dimension as the model’s output by a linear layer. $A_{i}$ denotes different attention calculation methods. This paper mainly uses four common attention mechanisms: Vanilla Attention, ProbSparse Attention, LSH Attention, and AutoCorrelation. AutoCorrelation is not, strictly speaking, part of the attention mechanism family. However, its effect is similar to or even better than attention mechanisms, so it is introduced into our model and involved in feature extraction.

Attention is the core building block of Transformer and is considered an essential tool for information capture in both CV and NLP domains. Many researchers have worked on designing more efficient attention, so many variants based on Vanilla Attention have been proposed in succession. The following briefly describes the four attention mechanisms used in our model.

#### 3.3.3. Vanilla Attention

Vanilla Attention was first proposed in the Transformer [3], and its input consists of three vectors: queries, keys, and values (Q,K,V), whose dimensions are $d_{k},d_{k},d_{v}$, respectively. Vanilla Attention is also known as Scaled Dot Product Attention because it is computed by dot product using Q and K and then scaled by $\sqrt{d_{k}}$. The specific calculation process is shown in Equation (3).

(3) $$A\left(Q,K,V\right)=\sigma^{†}\left(\frac{{\mathrm{QK}}^{⊤}}{\sqrt{d_{k}}}\right)V$$

Here, A denotes the attention or autocorrelation mechanism. $\sigma^{†}$ denotes the softmax activation function.

#### 3.3.4. ProbSparse Attention

This attention mechanism, first proposed in Informer, considers the attention coefficients’ sparsity and specifies the query matrix Q using the exact query sparsity measurement method (Algorithm 5). Equation (4) gives the ProbSparse Attention calculation method.

(4) $$A\left(Q,K,V\right)=\sigma^{†}\left(\frac{\tilde{Q}K^{⊤}}{\sqrt{d_{k}}}V\right)$$

Here, $\tilde{Q}$ is the sparse matrix obtained by the sparsity measure. The prototype of $\tilde{M}\left(q_{i},K\right)$ is Kullback–Leibler (KL) divergence, see Equation (5).

(5) $$KL\left(q\|p\right)=\ln\sum_{l=1}^{L_{K}}e^{\frac{q_{i}k_{l}^{⊤}}{\sqrt{d}}}-\frac{1}{L_{K}}\sum_{j=1}^{L_{K}}\frac{q_{i}k_{j}^{⊤}}{\sqrt{d}}-\ln L_{K}$$

**Algorithm 5** Explicit Query Sparisity Measurement**Require:**  Q,K**Ensure:**  $\tilde{Q}$
  1:**Define** $\tilde{M}\left(q_{i},K\right)=\max_{j}\left\{\frac{q_{i}k_{j}^{⊤}}{\sqrt{d_{K}}}\right\}-\frac{1}{L_{K}}\sum_{j=1}^{L_{K}}\frac{q_{i}k_{j}^{⊤}}{\sqrt{d_{K}}}$  2:**Define** $U={\mathrm{argTopu}}_{q_{i}\in \left[1,\cdots ,L_{Q}\right]}\left(\tilde{M}\left(q_{i},K\right)\right)$  3:**for** $u\in [1,\cdots ,L_{Q}]$ **do**  4:   **if** u∈U **then**  5:     ${\tilde{Q}}_{u,:}\leftarrow Q_{u,:}$  6:   **else**  7:     ${\tilde{Q}}_{u,:}\leftarrow 0$  8:   **end if**  9:
**end for**



#### 3.3.5. LSH Attention

Like ProbSparse Attention, LSH Attention also uses a sparsification method to reduce the complexity of Vanilla Attention. The main idea is that for each query, only the nearest keys are focused on, where the nearest neighbour selection is achieved by locally sensitive hashing. The specific attentional process of LSH Attention is given in Equation (6), where the hash function used is Equation (7):

(6) $$A\left(q_{i},K,V\right)=\sum_{j\in P_{i}}\frac{a(q_{i},k_{j})}{\sum_{l\in P_{i}}a\left(q_{i},k_{l}\right)}v_{j}$$

(7) $$h\left(x\right)=\arg\max\left(xR\|-xR\right)$$

where $P_{i}=\left\{j:h\left(q_{i}\right)=h\left(k_{j}\right)\right\}$ denotes the set of key vectors that the *i*-th query focuses on. $a\left(q_{i},k_{j}\right)=\exp\left(\frac{q_{i}k_{j}^{⊤}}{\sqrt{d}}\right)$ is used to measure the association of nodes *i* and *j*.

#### 3.3.6. AutoCorrelation

AutoCorrelation mechanisms are different from the types of attention mechanisms above. Whereas the self-attentive family focuses on the correlation between points, the AutoCorrelation mechanism focuses on the correlation between segments. Therefore, AutoCorrelation mechanisms are an excellent complement to self-attentive mechanisms.

(8) $$A\left(Q,K,V\right)=\sum_{\tau \in T}\mathrm{roll}\left(V,\tau \right)\cdot \sigma^{†}\left(R_{Q},K\left(\tau \right)\right)$$

(9) $$R_{Q},K\left(\tau \right)=\frac{1}{L}\sum_{t=1}^{L}Q_{t}K_{t-\tau }$$

(10) $$T=\left\{\tau_{1},\tau_{2},\cdots ,\tau_{k}\right\}={\mathrm{argTopk}}_{\tau \in \{1,\cdots ,L\}}\left(R_{Q},K\left(\tau \right)\right)$$

Equation (8) gives the procedure of calculating the AutoCorrelation mechanism, where Equation (9) is used to measure the correlation between two sequences, and τ denotes the order of the lag term. roll(V,τ) denotes the vector of τ-order lagged terms of vector V obtained in a self-looping manner. Equation (10) is the Topk algorithm used to filter the set T of *k* lagged terms with the highest correlation.

### 3.4. GAT Network

The Vanilla Transformer model embeds a Feedforward Network (FFN) [37] layer at the end of each encoder–decoder layer. The FFN plays a crucial role in mitigating token-uniformity inductive bias. Inductive bias can be considered a learning algorithm as a heuristic or “value” for selecting hypotheses in ample hypothesis space. For example, convolutional networks assume that information is spatially local, spatially invariant, and translational equivalent, so that the parameter space can be reduced by sliding convolutional weight sharing; recurrent neural networks assume that information is sequential and invariant to temporal transformations, so that weight sharing is also possible. Similarly, the attention mechanism also has some assumptions, such as the uselessness of some information. If the attention mechanism is stacked, some critical information will be lost, so adding a layer of FNN can somehow alleviate the accumulation of inductive bias and avoid network collapse. Of course, not only does the FFN layer have a mitigating effect, but we find that a similar effect can be achieved using a Graph Neural Network (GNN) [38,39,40]. Here, we use a two-layer GAT [41,42] network instead of the original FFN layer. The graph network has the property of aggregating the information of neighbouring nodes, i.e., through the aggregation of the graph network, each node will fuse some features of its neighbouring nodes. Additionally, we use random sampling to reduce the complexity. The reason is that our goal is not feature aggregation, but to mitigate the loss of crucial information. In particular, when the number of samples per node is 0, the graph network can be considered to ultimately degenerate into an FFN layer with a similar role to the original FFN.

Here, we model each token as a node in the graph and mine the dependencies between nodes using the graph attention algorithm. The input to GAT is defined as $H=\{{\vec{h}}_{1},{\vec{h}}_{2},\cdots ,{\vec{h}}_{N}\}$. Here, ${\vec{h}}_{i}\in R^{F}$ denotes the input vector of the *i*-th node, *N* denotes the number of nodes in the graph, and *F* denotes the dimensionality of the input vector. Through the computation of the GAT network, this layer generates a new set of node features $H^{\prime }=\left\{{\vec{h}}_{1}^{\prime },{\vec{h}}_{2}^{\prime },\cdots ,{\vec{h}}_{N}^{\prime }\right\}$. Similarly, here ${\vec{h}}_{i}^{\prime }\in R^{F^{\prime }}$ denotes the output vector of the *i*-th node, and $F^{\prime }$ denotes the dimensionality of the output vector.

**Figure 3:**
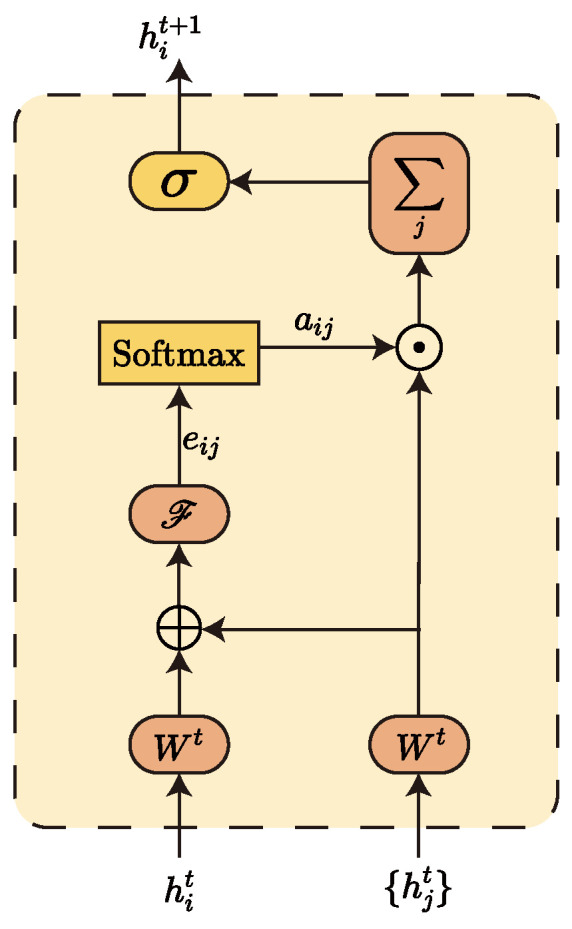
Feature aggregation process for node *i* in GAT network.

Figure 3 gives the general flow of information aggregation for a single node. Equation (11) is a concrete implementation of calculating the attention coefficient $e_{ij}$ for the *i*-th node and its neighbour node *j* one by one. Equation (12) is used to calculate the normalised attention factor $\alpha_{ij}$:

(11) $$e_{ij}=F\left(W{\vec{h}}_{i}\|W{\vec{h}}_{j}\right)$$

(12) $$\alpha_{ij}=\sigma^{†}\left(e_{ij}\right)=\frac{\exp(e_{ij})}{\sum_{k\in N_{i}}\exp\left(e_{ik}\right)}$$

Here, $N_{i}$ denotes the set of all neighbouring nodes of the *i*-th node, and W is a shared parameter for linear mapping of node features. F is a single-layer feedforward neural network for mapping the spliced high-dimensional features into a real number $e_{ij}$. $e_{ij}$ is the attention coefficient of node j→i, and $\alpha_{ij}$ is its normalised value.

Finally, the new feature vector ${\vec{h}}_{i}^{\prime }$ of the current node *i* is obtained by weighting and summing the feature vectors of each neighbouring node according to the calculated attention coefficients, where ${\vec{h}}_{i}^{\prime }$ records the neighbourhood information of the current node.

(13) $${\vec{h}}_{i}^{\prime }=\sigma \left(\sum_{j\in N_{i}}\alpha_{ij}W{\vec{h}}_{j}\right)$$

Here, σ represents applying a non-linear activation function logistic sigmoid at the end.

Furthermore, if information aggregation is accomplished through the *K* head attention mechanism, the final output vector can be obtained by taking the average.

(14) $${\vec{h}}_{i}^{\prime }=\sigma \left(\frac{1}{K}\sum_{k=1}^{K}\sum_{j\in N_{i}}\alpha_{ij}^{k}W^{k}{\vec{h}}_{j}\right)$$

## 4. Experiment

### 4.1. Dataset Description

To evaluate the Metaformer model, we conducted experiments on four popular real-world datasets encompassing energy, economy, disease, and transportation domains. The Electricity (https://archive.ics.uci.edu/ml/datasets/ElectricityLoadDiagrams20112014, accessed on 24 February 2023) dataset describes the hourly electricity consumption of 321 customers; the Exchange [43] dataset describes the daily exchange rates of eight countries; the Illness (https://gis.cdc.gov/grasp/fluview/fluportaldashboard.html, accessed on 24 February 2023) dataset is the weekly data of influenza-like illnesses recorded by the Centers for Disease Control; and the Traffic (http://pems.dot.ca.gov/, accessed on 24 February 2023) dataset describes the occupancy rate of roads in the San Francisco Bay area.

Table 1 shows detailed dataset statistics, where #Sample is the total number of samples, #Features is the number of features acquired per sampling, Period is the sampling period, and Span is the sampling time span.

**Figure 4:**
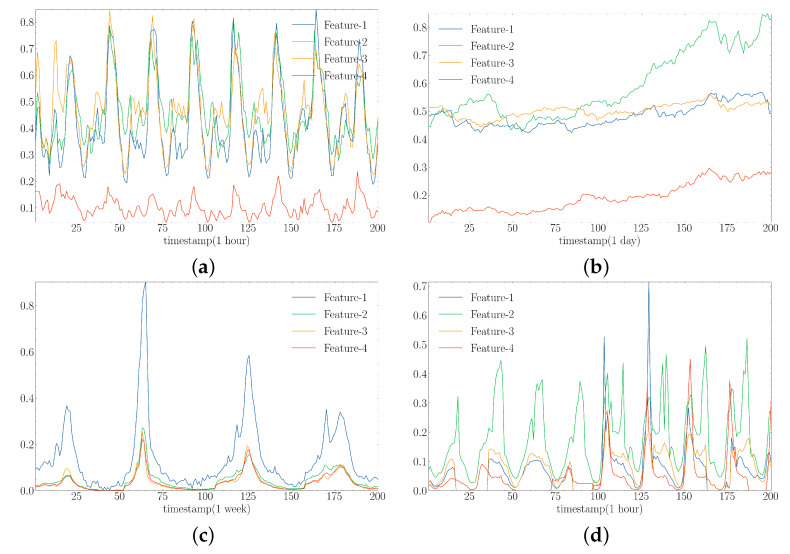
Variation of the four randomly selected normalised features in the dataset. (**a**) Electricity. (**b**) Exchange. (**c**) Illness. (**d**) Traffic.

Since the scale of each element in the dataset is not uniform, we need to normalise the data before formal training for the model to treat different features equally during training. Equations (15) and (16) are the normalisation and denormalisation calculation methods, respectively, where *X* denotes the original sampled dataset and $X^{*}$ denotes the normalised dataset. Figure 4 shows the variation of the four normalised features randomly selected from the four data sets.

(15) $$X^{*}=\frac{X-E(x)}{\sqrt{D(X)}}$$

(16) $$X=X^{*}\cdot \sqrt{D(X)}+E\left(X\right)$$

### 4.2. Comparison Experiments

#### 4.2.1. Baseline Models

To validate the predictive performance of our proposed model, we thoroughly compare it with some state-of-the-art time series prediction models, including Autoformer [33], Informer [32], Reformer [31], LogTrans [44], LSTNet [43], LSTM [24], and TCN [45]. Among them, Autoformer, Informer, Reformer, and LogTrans are all improved models based on Transformer. Autoformer uses an adaptive attention mechanism and dynamic feature transformation to adapt to different time steps and missing data, and can handle long sequences well. LogTrans is an autoregressive model that can take nonlinear and non-stationary data with good robustness and robustness by the logarithmic transformation of the input data. LSTM is a classical recurrent neural network model with a gating mechanism that can effectively deal with the forgetting problem of long-series data prediction. TCN is a convolutional neural network model that can handle the long-term dependence and nonlinear variation of long series by adding residual connections between the convolutional layers, and has high efficiency, good robustness, and small memory occupation.

#### 4.2.2. Experimental Setup

To standardise the sequence input length I=96 for comparison, we use a 7:1:2 ratio to split the Electricity, Exchange, and Traffic datasets into training, validation, and test sets, respectively, and set the prediction length O∈{96,192,336,720} accordingly. For the ILI dataset, we use a 6:2:2 split and set the prediction length O∈{24,36,48,60} accordingly. We set the dimensionality of the model to $d_{m}=512$ and use a hierarchical attention mechanism with four layers, which stacks AutoCorrelation, Vanilla Attention, LSH Attention, and ProbSparse Attention from top to bottom. The number of attention heads is set to 2. Additionally, to ensure comparability, we uniformly set the number of heads to 8 for the multi-headed attention mechanism in the other Transformer families involved in the comparison. In the GAT network, we use a two-layer architecture with a middle hidden layer dimension of 1024, and each node is assigned to have only one edge pointing to itself (self-loop graph). The sliding window size of the decoder’s moving average is set to 25, the number of encoder layers is set to N=2, and the number of decoder layers is set to M=1. We use MSE as the loss function and Adam as the optimiser with a learning rate of 0.0001. We train the model for 20 iterations, but employ an early termination strategy with a tolerance of 3.

#### 4.2.3. Experimental Result

**Figure 5:**
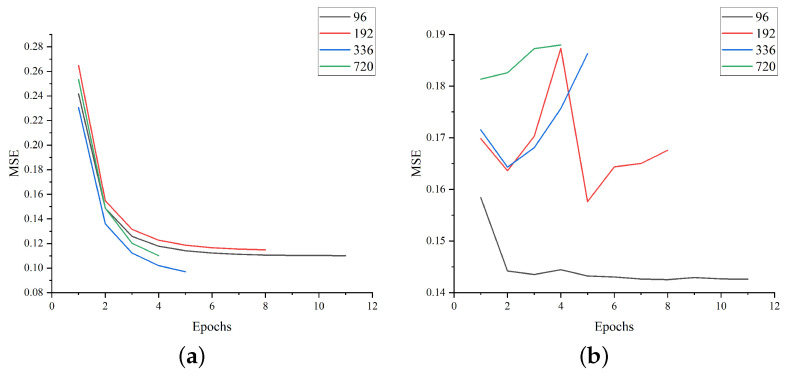

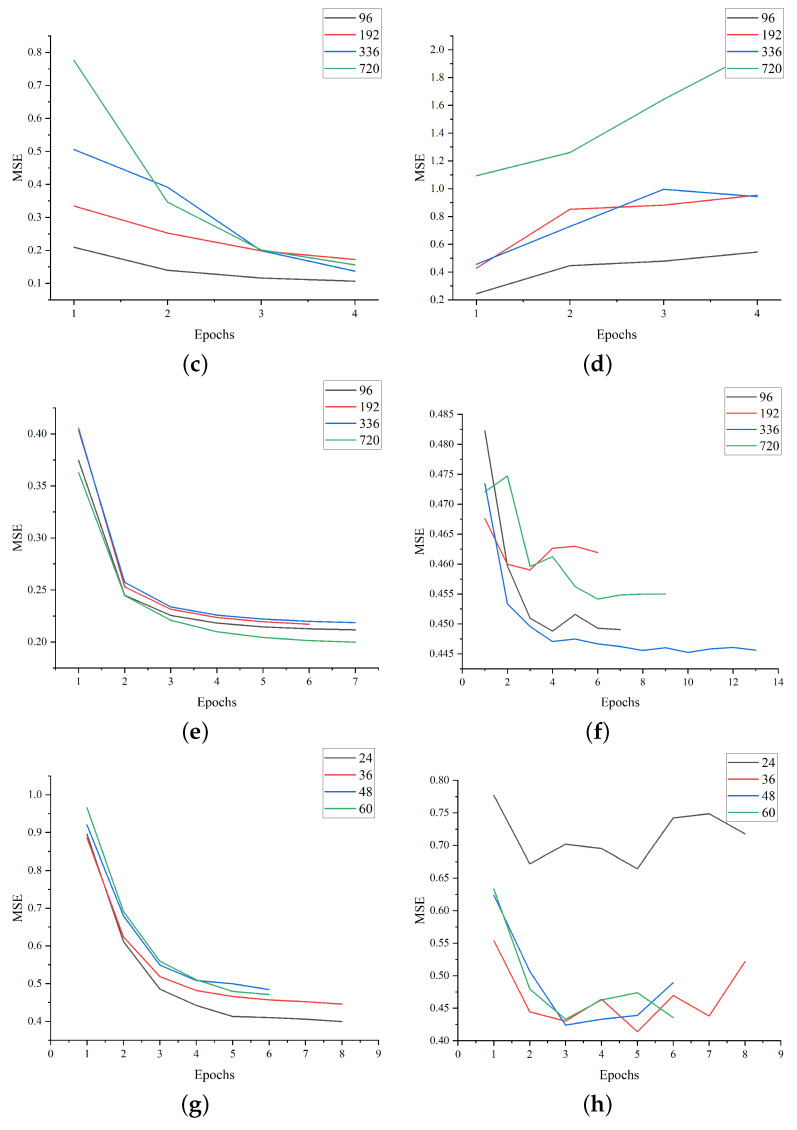
The MSE loss plots for the Metaformer model on the training and test sets for the four datasets. (**a**) Electricity (train), (**b**) Electricity (vali), (**c**) Exchange (train), (**d**) Exchange (vali), (**e**) Traffic (train), (**f**) Traffic (vali), (**g**) ILI (train), (**h**) ILI (vali).

Figure 5 shows the decreasing trend of the loss value in the training set and the loss value in the test set of our model during the training process. Table 2 presents an overall comparison between our model and other baseline models. The table shows that the Transformer-based model delivers significantly better predictions than other models. Autoformer performs well on several datasets and exhibits lower MAE and MSE values than other models. Informer is also a good model, but does not perform as well as Autoformer on some datasets, where LSTM and TCN generally exhibit higher MAE and MSE values. In contrast, our model achieves optimal or suboptimal accuracy levels for different prediction lengths on different datasets. Its overall performance is better than other baseline models, indicating that our model can satisfy most sequence prediction tasks.

### 4.3. Ablation Experiments

Additional ablation experiments were conducted to investigate further the impact of different graph structures in alleviating the inductive bias. Table 3 presents three different graph structures, where Meta-v1 indicates that all nodes in the graph use only a self-loop structure; Meta-v2 indicates that all nodes in the graph use full bi-directional connectivity; and Meta-v3 indicates that all nodes in the graph have a self-loop structure for each node, in addition to full bi-directional connectivity. Table 4 displays the performance of three variants of the Metaformer model on the four datasets.

As shown in Table 4, the Meta-v1 variant of the model, which uses only the self-loop graph, generally outperforms the other variants across multiple measures. This phenomenon may be because the self-loop edges are self-weighted, which is more effective in reducing the inductive bias of the attention mechanism in the Metaformer model by reinforcing the features of specific nodes. Conversely, adding a fully connected mechanism may further exacerbate the information perturbation. However, due to limited experimental resources, we cannot conduct a more in-depth study. In future work, we will further investigate how random sampling of neighbouring nodes, including more attention mechanisms, and the stacking order of these attention mechanisms affect the model’s performance.

## 5. Conclusions

This paper presents a redesigned sequence-to-sequence model based on the Transformer architecture. We draw inspiration from the sequence decomposition model of Autoformer and introduce a similar approach to separate trend and seasonal items. Additionally, we propose a hierarchical attention mechanism to address the problem of incomplete and insufficient information mining by multiple attention mechanisms in the Vanilla Transformer model. Our hierarchical attention mechanism employs different attention mechanisms simultaneously to ensure diversity in information mining. The hierarchical structure recursively passes information captured by lower-level attention upward, enabling interaction between multiple attention mechanisms and deepening the network’s understanding of more profound information. This mechanism is beneficial in capturing the metaphorical information present in both text and images. We also add a graph attention network to the model, allowing it to stand in a high-dimensional perspective to aggregate and mitigate the inductive bias of the information. Our experimental results demonstrate that our proposed model outperforms the baseline model across multiple datasets and significantly improves all evaluation metrics.

## Figures and Tables

**Table 1:** Overall statistical indicators for the selected dataset.

Dataset	#Sample	#Features	Period	Span
Electricity	26,304	321	hourly	2012–2014
Exchange	7588	8	daily	1990–2016
Illness	966	7	hourly	2002–2021
Traffic	17,544	862	weekly	2016–2018

**Table 2:** Multivariate long-term series prediction results for four datasets with an input length of I=96 and prediction length of O∈{96,192,336,720}. Lower MSE and MAE values indicate better results, and the best results are highlighted in bold.

Models	Metric	Electricity				Exchange				Traffic				ILI			
		96	192	336	720	96	192	336	720	96	192	336	720	24	36	48	60
**Metaformer**	**MSE**	**0.184**	**0.194**	**0.203**	**0.243**	**0.157**	**0.265**	**0.432**	**1.387**	**0.569**	**0.561**	**0.592**	**0.611**	**3.004**	**2.852**	**2.653**	**2.769**
	**MAE**	**0.297**	**0.305**	**0.319**	**0.347**	**0.29**	0.38	**0.483**	**0.924**	**0.345**	**0.347**	0.363	**0.368**	**1.193**	**1.142**	1.088	**1.085**
**Autoformer**	**MSE**	0.201	0.222	0.231	0.254	0.197	0.3	0.509	1.447	0.613	0.616	0.622	0.66	3.483	3.103	2.669	2.77
	**MAE**	0.317	0.334	0.338	0.361	0.323	**0.369**	0.524	0.941	0.388	0.382	**0.337**	0.408	1.287	1.148	**1.085**	1.125
**Informer**	**MSE**	0.274	0.296	0.3	0.373	0.847	1.204	1.672	2.478	0.719	0.696	0.777	0.864	5.764	4.755	4.763	5.264
	**MAE**	0.368	0.386	0.394	0.439	0.752	0.895	1.036	1.31	0.391	0.379	0.42	0.472	1.677	1.467	1.469	1.564
**Reformer**	**MSE**	0.312	0.348	0.35	0.34	1.065	1.188	1.357	1.51	0.732	0.733	0.742	0.755	4.4	4.783	4.832	4.882
	**MAE**	0.402	0.433	0.433	0.42	0.829	0.906	0.976	1.016	0.423	0.42	0.42	0.423	1.382	1.448	1.465	1.483
**LogTrans**	**MSE**	0.258	0.266	0.28	0.283	0.968	1.04	1.659	1.941	0.684	0.685	0.733	0.717	4.48	4.799	4.8	5.278
	**MAE**	0.357	0.368	0.38	0.376	0.812	0.851	1.081	1.127	0.384	0.39	0.408	0.396	1.444	1.467	1.468	1.56
**LSTNet**	**MSE**	0.68	0.725	0.828	0.957	1.551	1.477	1.507	2.285	1.107	1.157	1.216	1.481	6.026	5.34	6.08	5.548
	**MAE**	0.645	0.676	0.727	0.811	1.058	1.028	1.031	1.243	0.685	0.706	0.73	0.805	1.77	1.668	1.787	1.72
**LSTM**	**MSE**	0.375	0.442	0.439	0.98	1.453	1.846	2.136	2.984	0.843	0.847	0.853	1.5	5.914	6.631	6.736	6.87
	**MAE**	0.437	0.473	0.473	0.814	1.049	1.179	1.231	1.427	0.453	0.453	0.455	0.805	1.734	1.845	1.857	1.879
**TCN**	**MSE**	0.985	0.996	1	1.438	3.004	3.048	3.113	3.15	1.438	1.463	1.479	1.499	6.624	6.858	6.968	7.127
	**MAE**	0.813	0.821	0.824	0.784	1.432	1.444	1.459	1.458	0.784	0.794	0.799	0.804	1.83	1.879	1.892	1.918

**Table 3:** Three variants of Metaformer. ✓ and ✗ indicate that the specified structure was or was not used, respectively.

Models	Self-Loop	Full Connection
Meta-v1	✓	✗
Meta-v2	✗	✓
Meta-v3	✓	✓

**Table 4:** Performance of three variants of the Metaformer model on four datasets.

Models	Metric	Electricity				Exchange				Traffic				ILI			
		96	192	336	720	96	192	336	720	96	192	336	720	24	36	48	60
Meta-v1	MSE	0.184	0.194	0.203	0.243	0.157	0.265	0.432	1.387	0.569	0.561	0.592	0.611	3.004	2.852	2.653	2.769
	MAE	0.297	0.305	0.319	0.347	0.29	0.38	0.483	0.924	0.345	0.347	0.363	0.368	1.193	1.142	1.088	1.085
Meta-v2	MSE	0.195	0.197	0.212	0.233	0.245	0.702	0.973	1.222	0.607	0.588	0.59	0.617	3.195	3.438	3.318	3.075
	MAE	0.309	0.309	0.324	0.34	0.375	0.647	0.767	0.896	0.397	0.37	0.369	0.383	1.231	1.321	1.301	1.161
Meta-v3	MSE	0.196	0.199	0.203	0.236	0.268	0.706	0.963	1.768	0.615	0.596	0.593	0.621	3.643	3.289	2.967	3.317
	MAE	0.309	0.312	0.316	0.344	0.375	0.65	0.756	1.074	0.403	0.379	0.37	0.385	1.375	1.271	1.207	1.246

## Data Availability

Not applicable.
